# Statics-Based Model-Free Damage Detection under Uncertainties Using Modal Interval Analysis

**DOI:** 10.3390/ma13071567

**Published:** 2020-03-28

**Authors:** Sheng-En Fang, Ji-Yuan Huang

**Affiliations:** 1 School of Civil Engineering, Fuzhou University, Fuzhou 350108, China; n170520067@fzu.edu.cn; 2 National & Local Joint Engineering Research Center for Seismic and Disaster Informatization of Civil Engineering, Fuzhou University, Fuzhou 350108, China

**Keywords:** damage detection, uncertainties, modal interval analysis, classic interval analysis, response interval envelopes

## Abstract

Deterministic damage detection methods often fail in practical applications due to ever-present uncertainties. Moreover, vibration-based model updating strategies are easily affected by measurement noises and could encounter ill-conditioning problems during inverse solutions. On this account, a model-free method has been proposed combining modal interval analyses with static measurements. Structural geometrical dimensions, material parameters and external loads are expressed by interval variables representing uncertainties. Mechanical formulas for static responses are then extended to their interval forms, which are subsequently solved using classic interval and modal interval analyses. The analytical interval envelopes of static responses such as deflections and strains are defined by the interval solutions, and damage can be detected when the measured responses intersect the envelopes. By this approach, potential damage can be found in a fast and rough way without any inverse solution process such as model updating. The proposed method has been verified against both numerical and experimental reinforced concrete beams whose strains were taken as the desirable responses. It was found that the strain envelopes provided by modal interval analysis were narrower than those by classic interval analysis. Modal interval analysis effectively avoids the phenomenon of interval overestimation. In addition, the intersection point also identifies the current external load, providing a loading alarm for structures.

## 1. Introduction

Civil infrastructures will encounter performance deterioration after long-term service. To guarantee their safety, structural health monitoring systems have been installed for large-scale civil structures such as bridges in recent years [1,2]. Structural damage is expected to be detected and evaluated using different vibration-based approaches [3]. However, uncertainties always exist in civil structures, e.g., material variabilities, unknown boundary/connection conditions, measurement noises and temperature effects [4]. Due to this, probabilistic or nonprobabilistic theories should be involved in the damage detection process [5]. Probabilistic methods are mostly used with the aid of stochastic finite element algorithms [6], statistical pattern recognition [7] and Bayesian inference [8]. When sufficient statistical information on structural parameters and responses is not available or will incur high costs, uncertainty-based damage detection must rely on fuzzy theories [9,10] or interval analyses [11,12]. A fuzzy logic system can be defined to map structural parameters to responses, and damage is detected by fuzzy clustering [10]. But the primary limitation of fuzzy methods lies in the accurate definition of membership functions or grades. Moreover, a fuzzy reasoning process has a certain level of subjectivity, increasing uncertainty.

On the other hand, engineers are often concerned about the maximum and minimum responses in the civil engineering realm. Interval analyses address such concerns, and thus, have recently received considerable attention from researchers. Structural parameters and responses can be defined by interval numbers with upper and lower bounds [11,12,13]. Concretely, structural responses are determined by solving a set of linear interval equations [14]. Parameter intervals are then estimated through an inverse optimization process [15,16]. The optimization process incorporates a model updating strategy with interval algorithms [15]. Also, a two-step interval updating procedure can be used to obtain the intervals of structural parameters before and after damage [16]. A fuzzy inference system is available for system identification where interval modeling is employed to quantify uncertainties [17]. Alternatively, constraint satisfaction problems provide a nonupdating option for damage detection purposes after their combination with interval analysis [18]. But the primary drawback of classical interval analysis (CIA) comes from the phenomenon of interval overestimation after interval arithmetic [11,12]. Estimated parameter intervals are usually larger than real ones, which often leads to wrong solutions [19]. Furthermore, it is difficult to directly implement interval inverse optimization [20], which is often simplified and done within a deterministic framework [21]. In addition, how to judge the damage based on estimated parameter intervals is worthy of investigation.

To date, most damage detection methods have been implemented on structural vibrational data [3,22,23]. But traditional data in the frequency domain, e.g., frequencies and mode shapes, are susceptible to measurement noises [24,25] and environmental factors [26]. Modern techniques using elastic wave propagation with the aid of the spectral finite element method have also been investigated for the purpose of structural health monitoring [27,28]. Nevertheless, their performance is constrained by the material inhomogeneity or geometrical irregularities of a structure [28]. Hence, static measurements such as displacements that are more stable in field testing can also be used for detection [29,30]. Meanwhile, damage is generally estimated by solving inverse problems with the help of finite element model updating [31]. However, the problem of ill-conditioning could occur when dealing with complex structures. The model updating process becomes time-consuming, and thus, is not suitable for practical applications. A model-free damage detection strategy could avoid this drawback.

This study attempts to develop a model-free method for fast and robust damage detection of reinforced concrete structures considering different types of uncertainties. Structural parameters and external loads are defined as interval numbers. The structural equation of statics is extended to its interval form. To restrain interval overestimation, modal interval analysis (MIA) [32,33] is adopted for calculating the interval envelopes of strain measurements. Structural damage, as well as current external loads, can be predicted according to the intersection between the analytical envelope and measured strains. The detection results have been compared with those calculated using CIA.

## 2. Classical Interval Analysis

In the real number field of ℜ, an interval number $x^{I}$ can be defined on a set of real numbers and expressed as

(1) $$x^{I}=[\underline{x},\bar{x}]=\left\{x\in ℜ|\underline{x}\leq x\leq \bar{x}\right\}$$

where $\underline{x}$ and $\bar{x}$ represent the upper and lower bounds, respectively. The set of all closed intervals is given by I(ℜ). In the case of $\underline{x}=\bar{x}$, degrades into a point interval that is equal to a real number. The four arithmetic operations of interval numbers are quite different from classical mathematics [12]. The primary drawback of classic interval arithmetic operations is the phenomenon of interval overestimation.

In the engineering realm, functions, instead of numbers, are usually investigated. Suppose a continuous function f(x) ($x=(x_{1},\cdots ,x_{n})$) defined within the domain $X=\left(X_{1},\cdots ,X_{n}\right)$; the range of its joint expansion function *R_f_* is given as follows

(2) $$R_{f}\left(X_{1},\cdots ,X_{n}\right)=\left[\min\left(x,X\right)f\left(x_{1},\cdots ,x_{n}\right),\max\left(x,X\right)f\left(x_{1},\cdots ,x_{n}\right)\right]$$

Then, the response of f(x) can be defined as a rational interval extension function of fR(X), which means that $\left(x_{1},\cdots ,x_{n}\right)$ in f(x) are replaced by $\left(X_{1},\cdots ,X_{n}\right)$. The operator for real numbers is also replaced by the interval one for further calculation. In general, it is difficult to calculate $R_{f}$, whose partial information is lost in fR. Hence, the interval calculation result is often overestimated, namely:

(3) $$R_{f}\;\left(X_{1},\ldots ,X_{n}\;\right)\subseteq fR(X_{1},\ldots ,X_{n})$$

In general, *fR* represents an overestimation of *R_f_*, implying that the interval bounds estimated by *fR* are larger than the true values. In some cases, the calculation results are divergent. To give an example, the interval of a function $f(x_{1},x_{2})=x_{1}-x_{1}x_{2}$ is sought on $X_{1}=\left[1,2\right]$ and $X_{2}=\left[5,6\right]$. The CIA calculation process is illustrated as follows:

$$\begin{array}{ll} f(x_{1},x_{2}) & =x_{1}-x_{1}x_{2}\\ & =\left[1,2\right]-\left[1,2\right]\times \left[5,6\right]\\ & =\left[1,2\right]-\left[5,12\right]\\ & =\left[-11,-3\right] \end{array}$$

It was found that the interval estimated by CIA was larger than the exact solution of [−10, −4], which is also called an interval overestimation.

## 3. Modal Interval Analysis

The theory of MIA incorporates interval mathematics with modal logic [32,33], and thus, can be regarded as a theoretical extension of CIA. The modal predicate is used to semantically interpret intervals in order to avoid interval overestimation. Specifically, the real variables in a general function are substituted by interval variables. Then, MIA redefines the interval function through the modal predicate, and the target interval is calculated using modal arithmetic operations.

Given that a set of closed intervals I(ℜ)={[a, b]|a,b∈ℜ, a<b} in ℜ, an existential quantifier E, and a universal quantifier U, the modal interval of *X_i_* is defined as

(4) $$X_{i}:=\left(X_{i},QX_{i}\right)$$

where $X_{i}\in I\left(ℜ\right)$ gives logical expansion and $QX_{i}\in (E,U)$ is called “modal”.

In classic mathematics, different real numbers may have the same absolute value but opposite signs. Modal intervals possess a similar property, that is, two modal intervals may have the same closed interval on the real axis, but opposite modes. Here, the above two quantifiers of E and U are used to express the set of modal intervals

(5) $$I^{*}\left(ℜ\right):=\left\{X_{i},\left\{E,U\right\}|X_{i}\in I\left(ℜ\right)\right\}$$

To be specific, modal intervals can be divided into two categories according to the relationship between the upper bound *a* and the lower bound *b*:

(6) $$\mathrm{Prop}\left(X_{i},QX\right):=\left(X_{i},E\right)=\left(\left[a,b\right],E\right)\in I^{*}\left(ℜ\right)\left(a<b\right)$$

which is named “existence interval” or “proper interval”.

(7) $$\mathrm{Impr}\left(X_{i},QX\right):=\left(X_{i},U\right)=\left(\left[a,b\right],U\right)\in I^{*}\left(ℜ\right)\left(a>b\right)$$

which is named “universal interval” or “improper interval”.

The transform between proper and improper intervals can be achieved by a dual operator

(8) $$\mathrm{Dual}\left(\left[a_{1},a_{2}\right]\right)=\left[a_{2},a_{1}\right]$$

The dual operation interchanges the upper and lower bounds of an interval, thereby transforming an improper interval to a proper one and vice versa. In Equation (8), the original interval of $\left[a_{1},a_{2}\right]$ has been transformed to $\left[a_{2},a_{1}\right]$ after the dual operation. After applying the dual operation to a continuous function *f*, two semantic functions, f* and f**, are obtained to provide logic meaning for interval calculation.

(9) $$\begin{array}{ll} f^{*}\left(X\right) & =\vee \left(a_{p},X_{p}\right)\wedge \left(a_{i},X_{i}\right)\left[f\left(a_{p},a_{i}\right),f\left(a_{p},a_{i}\right)\right]\\ & =\left[\min\left(a_{p},X_{p}\right)\max\left(a_{i},X_{i}\right)f\left(a_{p},a_{i}\right),\max\left(a_{p},X_{p}\right)\min\left(a_{i},X_{i}\right)f\left(a_{p},a_{i}\right)\right] \end{array}$$

(10) $$\begin{array}{ll} f^{**}\left(X\right) & =\wedge \left(a_{i},X_{i}\right)\vee \left(a_{p},X_{p}\right)\left[f\left(a_{p},a_{i}\right),f\left(a_{p},a_{i}\right)\right]\\ & =\left[\max\left(a_{i},X_{i}\right)\min\left(a_{p},X_{p}\right)f\left(a_{p},a_{i}\right),\min\left(a_{i},X_{i}\right)\max\left(a_{p},X_{p}\right)f\left(a_{p},a_{i}\right)\right] \end{array}$$

where symbols ∨ and ∧ denote the operation of taking the upper and lower bounds, respectively; $\left[f\left(a_{p},a_{i}\right),f\left(a_{p},a_{i}\right)\right]$ is a point interval; $\left(a_{p},a_{i}\right)$ corresponds to the modal interval $X=\left(X_{p},X_{i}\right)$; $X_{p}$ denotes a subvector in the proper subinterval of *X*, while $X_{i}$ represents a subvector in the improper subinterval of *X*.

With f* and f**, the four arithmetic operations of MIA can be carried out with the detailed descriptions in [32,33]. Then, back to the example of $f(x_{1},x_{2})=x_{1}-x_{1}x_{2}$ in Section 2, MIA gives the exact solution, i.e., [−10, −4].

## 4. Damage Detection Strategy

Online damage detection ability is essential for a structural health monitoring system. However, so far, in practice, the online monitoring of civil structures still focuses on whether a monitoring item exceeds the threshold defined by design codes or engineers. Accurate detection is presently impractical due to sensors limitations, measurement noises, environmental influence, etc. Therefore, an exploratory and rough damage evaluation is more realistic for on-site judgment. Damage detection strategies should be simple, using structural responses that are easy to obtain in the field.

Displacements and strains are two common measurement items for monitoring and regularly inspecting real-world civil infrastructures. For instance, the relatively large deflections of a bridge girder imply the potential loss of bending stiffness. Large strains indicate the possible sprout or appearance of cracks in concrete. Accordingly, the relationship between structural physical properties and displacements/strains can be correlated and then incorporated with interval analyses for the purpose of fast damage detection. Suppose a single-span simply supported uniform beam under small elastic deformations; its sectional deflection Δ(l) along the beam length has the expression as follows

(11) $$\Delta \left(l\right)=\frac{M\left(l\right)}{EI}$$

where M(l) denotes the moment of the cross-section at the coordinate of *l*, *E* denotes Young’s modulus, and *I* is the cross-sectional moment of inertia. M(l), *E,* and *I* are replaced by interval numbers if uncertainties exist in the external loads, the material properties, and the geometrical dimensions of the beam. After that, Equation (11) is calculated using CIA or MIA to obtain the interval envelopes of Δ(l), which are oblique lines for elastic beams. When measured deflections stay within the envelopes, the beam is assumed to be intact. Once measured deflections intersect the envelopes, damage could occur, which gives a rough judgment.

A similar procedure can also be used for strain measurements. For the previous beam, the strain at the bottom edge of an arbitrary cross-section is written as

(12) $$\varepsilon \left(l\right)=\frac{M\left(l\right)}{WE}$$

where *W* denotes the resistance moment of the cross-section. Again, Equation (12) can be extended to an interval form, and the interval envelopes of ε(l) can be established using CIA or MIA. The intersection of measured strains and interval envelopes implies the occurrence of damage.

The application of strain measurements is illustrated in the subsequent case studies. It is noted that the proposed damage detection strategy is easy to comprehend and handle for field engineers. The detection relies on forward solution of structural mechanical formulas, instead of inverse solutions using numerical models. The solution efficiency is highly improved. Uncertainties embedded in structural measurements are taken into account by interval analysis. The occurrence of damage is now determined by the intersection of the measurements and the interval envelopes. Thereby, damage can effectively and efficiently be detected. The strategy is suitable for fast judgment calls during accidents. A complex detection procedure is difficult to perform under such circumstances.

## 5. A Numerical Reinforced Concrete Beam

**Figure 1:**
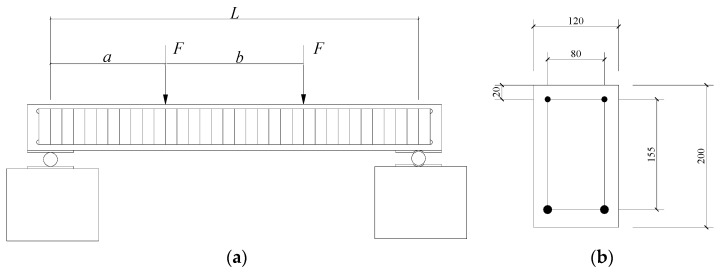
Schematic Diagram of Reinforced Concrete Beam: (**a**) elevation view; (**b**) cross section (Unit: mm).

A numerical simply supported beam was first used for validation, as shown in Figure 1. The beam was subjected to two identical concentrated forces, marked as *F*. The nominal elastic moduli of concrete and steel bars were *E*_c_ = 31GPa and *E_s_* = 200 GPa, respectively. The resistance moment at the bottom edge of the beam was *W* = 9.1 × 10^−4^ m^3^ after considering the effects of the concrete and steel bars. The strain at the bottom edge of the cross-sections within the pure bending segment can be calculated using the following formula:

(13) $$\begin{array}{ll} \varepsilon & =\frac{M}{WE}\\ & =\frac{F\left[L-\left(a+b\right)\right]+F\left[b+L-\left(a+b\right)\right]-Fb}{WE} \end{array}$$

The nominal load distances of *a* and *b* were set to 0.5 and 0.6 m, respectively. The load increment at each loading step was designed as 1 kN. Uncertainties were assumed to exist in *a*, *b*, *E*_c_, *W* and *F*, which had the intervals of a=[0.49,0.51] m, b=[0.59,0.61] m, $E_{c}=\left[28,34\right]$ GPa, $W=\left[8.1,10.1\right]\times 10^{-4}$ m^3^ and [0.95F,1.05F] kN. After that, Equation (13) was extended to its interval form, and the strain corresponding to each load step was calculated using CIA and MIA. Damage was assumed to happen when *F* arrived at 7 kN, since this load step $E_{c}$ was designed to decrease 8 GPa to simulate the loss of bending stiffness due to cracking.

**Figure 2:**
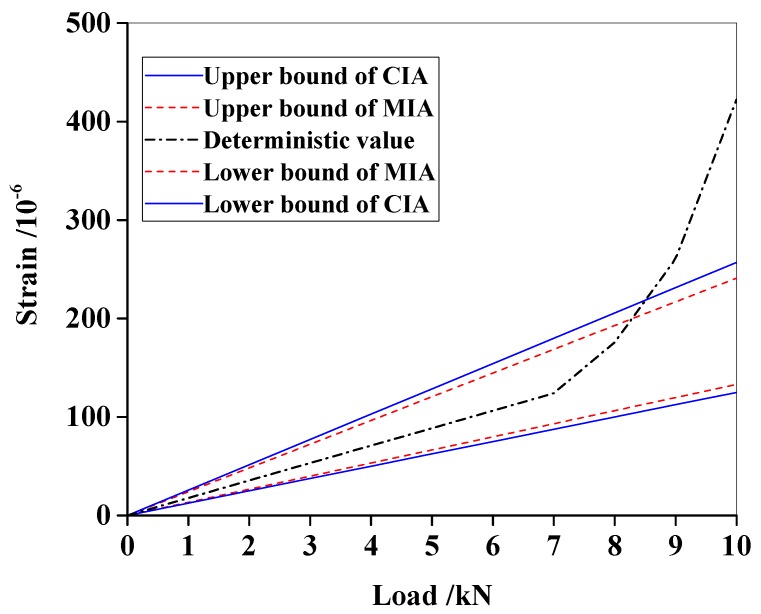
Strain envelopes of the numerical beam.

Table 1 shows that the intervals calculated by MIA are always smaller than those by CIA, proving that MIA can effectively avoid the phenomenon of interval overestimation. The deterministic strain calculated using Equation (13) intersects the intervals at the ninth load step, implying the occurrence of damage. For comparison, the strain envelopes are illustrated in Figure 2. It is observed that an inflection point appears at *F* = 7 kN, which accords with the deterministic damage assumption. However, in practice, such inflection could be caused by uncertainties or material defects, especially resulting in unstable strain measurements. This inflection is not reliable for damage detection. Therefore, the intersection between the measurement curve and the interval envelopes offers confirmation of damage occurrence. After the deterministic strain curve intersects the upper bound of MIA when *F* = 8.3 kN, it intersects the upper bound of CIA when *F* = 8.5 kN. Hence, MIA identifies the damage occurrence earlier than CIA, which could be an advantage in practice.

The Chinese code [34] offers the cracking load formula of a reinforced concrete beam:

(14) $$M_{\mathrm{cr}}=\frac{\gamma_{m}\;f_{\mathrm{tk}}I_{0}}{h-y_{0}}$$

(15) $$I_{0}=\left(0.083+0.19\alpha_{E}\rho \right)bh^{2}$$

(16) $$y_{0}=\left(0.5+0.425\alpha_{E}\rho \right)h$$

where *M_cr_* denotes the cracking moment, $y_{0}$ gives the distance of the cross-sectional center of gravity to the compression or tensile edge, $I_{0}$ is the moment of inertia with respect to the cross-sectional axis of the center of gravity, *b* and *h* are the width and height of the beam cross-section, respectively, $\gamma_{m}$ denotes a plasticity coefficient corresponding to cross-sectional resistance, *f_tk_* is the axial tensile strength of concrete, and ρ represents the reinforcement ratio and $\alpha_{E}=E_{s}/E_{c}$.

For the numerical beam, its analytical cracking moment was $M_{cr}=3128.5$N·m. The corresponding cracking load of $F_{\mathrm{cr}}=7.2$kN was close to that estimated by MIA.

## 6. An Experimental Reinforced Concrete Beam

### 6.1. Experimental Observations and Damage Detection

**Figure 3:**
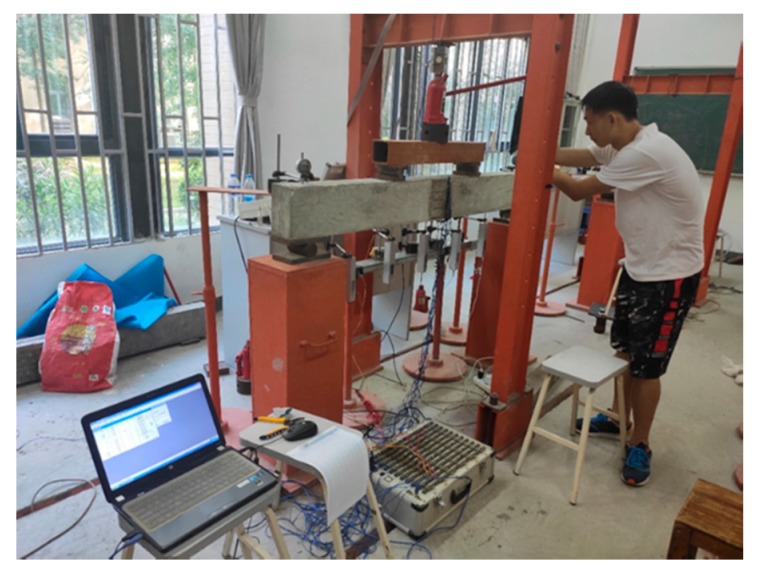
Laboratory static testing of the experiment beam.

**Figure 4:**
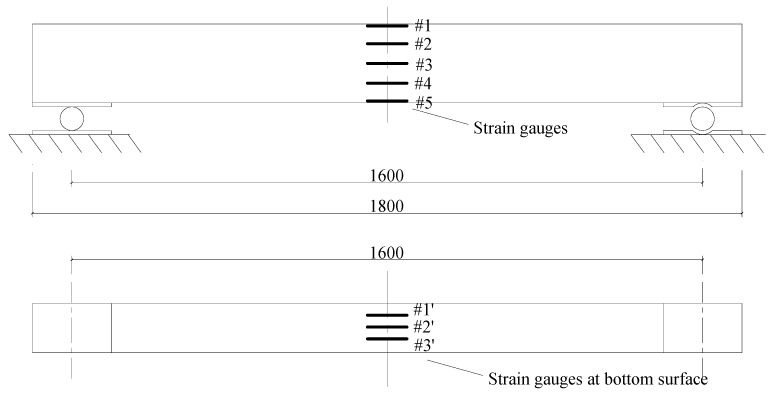
Strain gauge layout of the experimental beam (unit: mm).

**Figure 5:**
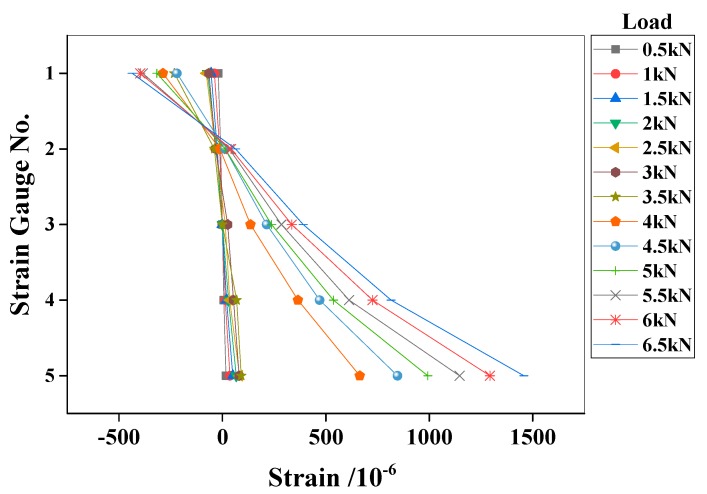
Plane cross-section assumption of the experimental beam.

An experimental reinforced concrete beam (Figure 3) was further tested to validate the proposed method. The geometrical dimensions and material properties of the experimental beam were identical to those of the numerical beam. Eight strain gauges were mounted on the concrete surface at the mid-span of the beam, as shown in Figure 4. Five of them (#1–#5) were mainly used to check the plane cross-section assumption of the beam. The left three strain (#1’–#3’) gauges on the bottom surface were used to detect the initial cracking of concrete. The applied load was provided by a hydraulic jack using a loading scheme similar to that of the numerical beam (Figure 1). The strains were continuously recorded with a time interval of 3 s during the test. The beam stayed at its elastic state until the load *F* reached 6.5 kN, because the plane cross-section assumption was confirmed by the actual measurements shown in Figure 5.

When the experimental load arrived at *F* = 4.5 kN, the measurements of the three strain gauges (#1’, #2’ and #3’) began to fluctuate. A tiny crack was visually found in the region near to these gauges. It was predicted that this crack was caused by the concrete defects during casting and curing, because it grew slowly during the test. The fluctuation became more serious for the side strain gauges of #1’, #3’ and #5 as the load reached around 5.5 kN. Their measured strains showed reductions at their points of inflection. At this moment, the tensile stress inside the concrete was partly released owing to the cracking of the concrete defects. The missing tensile stress was taken over by the tensile steel bars. Therefore, the strains nearby the defects decreased, resulting in a magnitude reduction of #1’, #3’ and #5. Their strains restarted increasing after *F* = 6 kN. Such a serious fluctuation was not observed in #2’ because the defect cracks mainly concentrated at the corners of the cross-sections.

**Figure 6:**
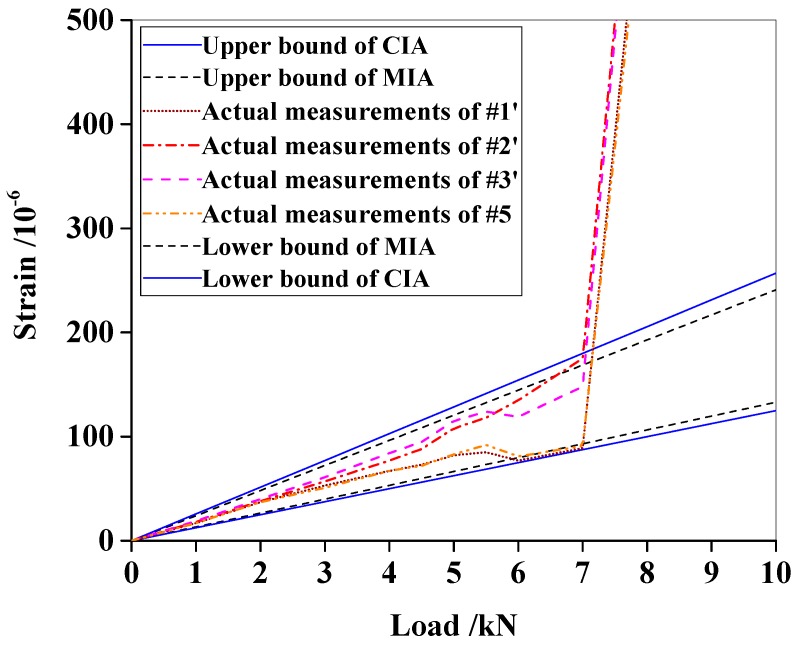
Strain interval envelopes of the experimental beam.

When the load reached around 7 kN, a visual main crack appeared at the beam bottom and grew rapidly with the increase of the load. The measured strains also went so fast that they suddenly intersected the interval envelopes, as shown in Figure 6. The main crack defined the cracking load. Therefore, the cracking load was determined to be 7 kN, which was close to the analytical solution of kN calculated using Equations (14), (15) and (16). Figure 6 also shows that MIA gives smaller interval predictions than CIA. For instance, the measured strain of #2’ successively intersects with the two envelopes, which means the actual strain exceeds the intervals considering the uncertainties. This observation proves that MIA can detect cracks earlier than CIA.

### 6.2. Discussions

Equation (13) involves simple mechanical principles that are easy to handle and calculate, which facilitates the easy implementation of fast field damage detection. This mechanical formula replaces commonly-used finite element models of structures. Its solution is a forward process which is more likely to be accepted by engineers who might not have adequate theoretical knowledge about damage detection.

For the numerical beam, the strain measurements were not polluted by noises, and thus, are clear (Figure 2). The initial damage occurrence could be determined by the inflection point at 7 kN. Therefore, one may question why damage was not detected according to the inflection points of the deterministic curves. The answer lies in the difference between theory and practice. It is known that experimental strain measurements always contain fluctuations caused by measurement noises and structural defects (Figure 6). Hence, it is difficult to find true inflection points. In addition, uncertainties in structural parameters and external loads cannot be embodied in deterministic curves. Response interval envelopes provide confidence intervals which do not preclude such uncertainties. The fluctuations of deterministic curves are embraced in the envelopes, and thus, will not be considered as damage. Thereby, false damage detection can be avoided to some extent.

Intervals also provide the maximum/minimum allowable values for response measurements under different loads. The intersection between envelopes and deterministic measurement curves indicates the response breaks through the predefined boundaries. Damage and current external loads can be determined at the intersection moment. On the other hand, the two beam examples showed that the cracking loads estimated by CIA were slightly behind those given by MIA (Figure 2 and Figure 6). This phenomenon is actually a prediction lag due to interval overestimation, which could be enlarged in practical applications because the beam examples had low-level uncertainties.

The successful validation of the proposed method reveals its potential use in more complex real-world civil structures. For instance, static testing should be regularly performed on bridges after long-term service in order to evaluate their condition. The static deflections and strains of bridge components such as girders are measured during testing. The measurements can be compared with the analytical interval envelopes of the intact components for fast judgment of damage occurrence.

## 7. Conclusions

A model-free damage detection method has been proposed based on interval analyses of static strain measurements. Finite element models are substituted by mechanical formulas that are easy to calculate, and inverse solutions are no longer required for structural parameter estimation. Theoretical formulas for strain calculation can be extended to interval forms by replacing deterministic variables with interval ones, which express uncertainties in geometrical dimensions, material properties and external loads. The strain interval envelopes are then calculated using MIA and CIA. Damage occurrence is confirmed once measured strain curves exceed the envelopes. The intersection moment also indicates the current external loads. The numerical and experimental beam examples have shown that MIA can effectively avoid interval overestimations and produce narrower envelopes than CIA. Thereby, damage can be detected earlier, which is an advantage for practical applications.

## Figures and Tables

**Table 1:** Strain predictions of the numerical beam.

Load Step	*F*/kN	ε by MIA		ε by CIA		ε_d_
		Lower Bound	Upper Bound	Lower Bound	Upper Bound	
1	1	13.3	24.1	12.5	25.7	17.7
2	2	26.6	48.2	25	51.4	35.4
3	3	39.9	72.3	37.5	77.1	53.2
4	4	53.2	96.4	50	102.8	70.9
5	5	66.5	120.5	62.5	128.5	88.6
6	6	79.8	144.6	75	154.2	106.3
7	7	93.1	168.7	87.5	179.9	124.1
8	8	106.4	192.8	100	205.6	175.8
9	9	119.7	216.9	112.5	231.3	260.3

Note: ε_d_ denotes the deterministic strains calculated using Equation (13).

## References

[1] Wenzel H. (2009). Health Monitoring of Bridges.

[2] Xu Y.L., Xia Y. (2012). Structural Health Monitoring of Long-Span Suspension Bridges.

[3] Yan Y.J., Cheng L., Wu Z.Y., Yam L.H. (2007). Development in vibration-based structural damage detection technique. Mech. Syst. Signal Process..

[4] Biondini F., Frangopol D.M. (2016). Life-cycle performance of deteriorating structural systems under uncertainty: Review. J. Struct. Eng. ASCE.

[5] Simoen E., De Roeck G., Lombaert G. (2015). Dealing with uncertainty in model updating for damage assessment: A review. Mech. Syst. Signal Process..

[6] Xu H., Cheng L., Su Z.Q., Guyader J.L. (2013). Damage visualization based on local dynamic perturbation: Theory; application to characterization of multi-damage in a plane structure. J. Sound Vib..

[7] Ren W.X., Lin Y.Q., Fang S.E. (2011). Structural damage detection based on stochastic subspace identification; statistical pattern recognition: I. Theorysmart Mater. Struct..

[8] Nichols J.M., Moore E.Z., Murphy K.D. (2011). Bayesian identification of a cracked plate using a population-based Markov Chain Monte Carlo method. Comput. Struct..

[9] Chandrashekhar M., Ganguli R. (2009). Damage assessment of structures with uncertainty by using mode-shape curvatures; fuzzy logic. J. Sound Vib..

[10] Taha R.M.M., Lucero J. (2005). Damage identification for structural health monitoring using fuzzy pattern recognition. Eng. Struct..

[11] Jaulin L., Kieffer M., Didrit O., Walter E. (2001). Applied Interval Analysis.

[12] Moore R., Kearfott R., Cloud M. (2009). Introduction to Interval Analysis.

[13] Fang S.E., Zhang Q.H., Ren W.X. (2015). An interval model updating strategy using interval response surface models. Mech. Syst. Signal Process..

[14] Rao S.S., Berke L. (1997). Analysis of uncertain structural systems using interval analysis. AIAA J..

[15] Gabriele S., Valente C., Brancaleoni F. (2007). An interval uncertainty based method for damage identification. Key Eng. Mater..

[16] Wang X.J., Yang H.F., Wang L., Qiu Z.P., Qiu Z.P. (2012). Interval analysis method for structural damage identification based on multiple load cases. J. Appl. Mech..

[17] Zhu F.T., Wu Y.J. (2014). A rapid structural damage detection method using integrated ANFIS; interval modeling technique. Appl. Soft Comput..

[18] Garcia O., Vehi J., Matos J.C., Casas J.R. (2008). Structural assessment under uncertain parameters via interval analysis. J. Comput. Appl. Math..

[19] Khodaparast H.H., Mottershead J.E., Badcock K.J. (2011). Interval model updating with irreducible uncertainty using the Kriging predictor. Mech. Syst. Signal Process..

[20] Hansen E., Walster G.W. (2004). Global Optimization Using Interval Analysis.

[21] Li S.L., Li H., Ou J.P. Model updating for uncertain structures with interval parameters. Proceedings of the Asia-Pacific Workshop on Structural Health Monitoring.

[22] Zou Y., Tong L., Steven G.P. (2000). Vibration-based model-dependent damage (delamination) identification; health monitoring for composite structures-a review. J. Sound Vib..

[23] Jassim Z.A., Ali N.N., Mustapha F., Jalil N.A.A. (2013). A review on the vibration analysis for a damage occurrence of a cantilever beam. Eng. Fail. Anal..

[24] Baneen U., Kinkaid N.M., Guivant J.E., Herszberg I. (2012). Vibration based damage detection of a beam-type structure using noise suppression method. J. Sound Vib..

[25] Viola E., Miniaci M., Fantuzzi N., Marzani A. (2015). Vibration analysis of multi-stepped; multi-damaged parabolic arches using GDQ. Curved Layer. Struct..

[26] Hios J.D., Fassois S.D. (2014). A global statistical model based approach for vibration response-only damage detection under various temperatures: A proof-of-concept study. Mech. Syst. Signal Process..

[27] Ostachowicz W., Kudela P., Krawczuk M., Zak A. (2012). Guided Waves in Structures for SHM: The Time-Domain Spectral Element Method.

[28] Miniaci M., Mazzotti M., Radzieński M., Kudela P., Kherraz N., Bosia F., Pugno N.M., Ostachowicz W. (2019). Application of a laser-based time reversal algorithm for impact localization in a stiffened aluminum plate. Front. Mater..

[29] Ren W.X., Fang S.E., Deng M.Y. (2011). Response surface based finite element model updating using structural static responses. J. Eng. Mech. ASCE.

[30] Chen S.Z., Wu G., Feng D.C. (2019). Damage detection of highway bridges based on long-gauge strain response under stochastic traffic flow. Mech. Syst. Signal Process..

[31] Friswell M.I. (2007). Damage identification using inverse methods. Philos. Trans. R. Soc..

[32] Gardeñes E., Sainz M., Jorba L., Calm R., Estela R., Mielgo H., Trepat A. (2001). Model Intervals. Reliab. Comput..

[33] Sainz M.A., Armengol J., Calm R., Herrero P., Jorba L., Vehi J. (2014). Modal Intervals.

[34] SL/T191-2008 (2009). Design Code for Hydraulic Concrete Structures.

